# Supplementary material for: Contact lenses contamination by Acanthamoeba spp. in Upper Egypt
Source: PLoS One. 2021 Nov 15;16(11):e0259847. doi: 10.1371/journal.pone.0259847 (PMC8592476; doi:10.1371/journal.pone.0259847)
Supplement: S2 File — (PDF) [file pone.0259847.s002.pdf]

PONE-D-21-09515R1 (Contact lenses contamination by *Acanthamoeba* spp. in Upper Egypt)

Sequencing data

S2. Sample 68 (JDP1, 405 BP):

CCGAATACATTAGCATGGGATAATGGAATAGGACCCTGTCCTCCTATTTTC
AGTTGGTTTTGGCAGCGCGAGGACTAGGGTAATGATTAATAGGGATAGTT
GGGGGCATTAATATTTAATTGTCAGAGGTGAAATTCTTGGATTTATGAAA
GATTAACTTCTGCGAAAGCATCTGCCAAGGATGTTTTCATTAATCAAGAA
CGAAAGTTAGGGGATCGAAGACGATCAGATACCGTCGTAGTCTTAACCAT
AAACGATGCCGACCAGCGATTAGGAGACGTTGAATACAAAACACCACCA
TCGGTGCGGTCGTCCTTGGCGTCTCGGTTTCGGCCGGGGCGCGGGGATGG
CTTAGCCCGGTGGCACCGGTGAATGACTCCCTA

Similarity on Genebank: *Acanthamoeba* sp. isolate T4 clone ac2t4 (99.74 %) with 100% query cover.
