# Supplementary material for: Contact lenses contamination by Acanthamoeba spp. in Upper Egypt
Source: PLoS One. 2021 Nov 15;16(11):e0259847. doi: 10.1371/journal.pone.0259847 (PMC8592476; doi:10.1371/journal.pone.0259847)
Supplement: Attachment — Submitted filename: Response to Reviewers.doc [file pone.0259847.s003.doc]

**Reply to the reviewers’ comments**

| **Reviewer Number** | **Original comments of the reviewer** | **Reply by the author(s)** | **Changes done on page number and line number** |
| --- | --- | --- | --- |
| Reviewer #1 | 1- What kind of contact lens were assayed contamination by acanthamoeba? 2- Were the lenses solution related to the used lenses or new lenses or none of them? 3- Why the number of new lenses is not considered as the same as used lenses? | 1-Cosmetic (colored, not intended for medical use) lenses were assayed. Manufacture names were not recorded to avoid legal sequences.  2-lens solutions were related to use lenses.  3-New samples were easier to obtain and the surprising results that they might be contaminated made us explore more companies. | 1- Page (5), line (117) and (118). 2- Page (5), line (119). 3- Page (5), line (118) and (119). |
| Reviewer #1 | No comments | ___________________________________ | _____________________ |
